# Coevolution of *aah*: A *dps*-Like Gene with the Host Bacterium Revealed by Comparative Genomic Analysis

**DOI:** 10.1100/2012/504905

**Published:** 2012-02-01

**Authors:** Liyan Ping, Matthias Platzer, Gaiping Wen, Nicolas Delaroque

**Affiliations:** ^1^Department of Bioorganic Chemistry, Max Planck Institute for Chemical Ecology, Hans-Knöll-Straße 8, 07745 Jena, Germany; ^2^Genome Analysis, Leibniz Institute for Age Research-Fritz Lipmann Institute, Beutenbergstraße 11, 07745 Jena, Germany

## Abstract

A protein named AAH was isolated from the bacterium *Microbacterium arborescens* SE14, a gut commensal of the lepidopteran larvae. It showed not only a high sequence similarity to Dps-like proteins (DNA-binding proteins from starved cell) but also reversible hydrolase activity. A comparative genomic analysis was performed to gain more insights into its evolution. The GC profile of the *aah* gene indicated that it was evolved from a low GC ancestor. Its stop codon usage was also different from the general pattern of Actinobacterial genomes. The phylogeny of *dps*-like proteins showed strong correlation with the phylogeny of host bacteria. A conserved genomic synteny was identified in some taxonomically related Actinobacteria, suggesting that the ancestor genes had incorporated into the genome before the divergence of Micrococcineae from other families. The *aah* gene had evolved new function but still retained the typical dodecameric structure.

## 1. Introduction

A protein isolated in 1992 from *Escherichia coli *cells can bind DNA *in vitro* and was proposed to protect DNA from oxidative damage [1]. Its name DNA-binding protein from starved cell (Dps) has been widely accepted. However, some proteins belonging to the same family, which was isolated earlier, do not bind DNA, that is, the 4D antigen of *Treponema pallidum* isolated in 1987 [2], which was also known as TpF1 [3] and C1-5 [4]. They were proposed to play an important structural role in the outer membrane of the bacteria. Immunoelectron microscopy confirmed its surface localization [2], but it precipitated differently from other membrane proteins in ultracentrifugation [5]. 

**Figure 1:**
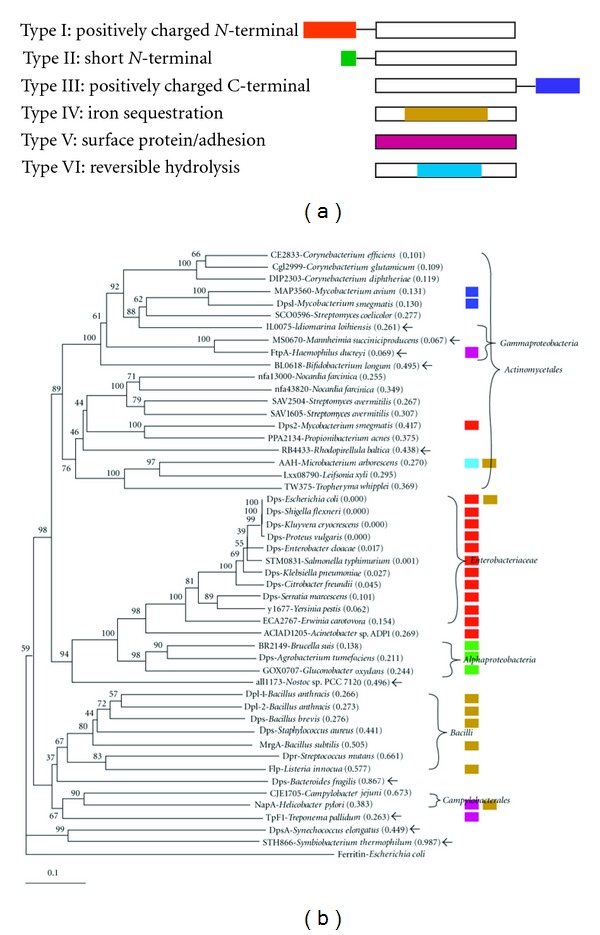
Functional domain, taxonomy, and sequence similarity of Dps-like proteins. (a) Diagram display of the functional domains of Dps-like proteins. Domains are colored as described on the left. The open blank bar depicts the protein including ferroxidase center. (b) Cladogram of some Dps-like proteins with branch lengths in accordance with their relative evolutionary distance. The unit is shown on the bottom. Another set of independently calculated distance values was given in parentheses. The bootstrap values (%) of 1000 repeats were labelled on the knots. Protein names were followed by the host species. Putative proteins were labelled with the gene tag in corresponding genome projects. The ferritin from *E. coli* was chosen as out-group. The classification of the corresponding hosts was shown on right. Those proteins whose sequence similarities and host phylogeny do not match were marked by arrows. The protein domains were shown as squares with the same color in (a). The protein IDs were listed in the Supplementary Material available online.

The DNA-binding activity of the *E. coli* Dps protein is due to a nonspecific interaction between the positively charged N-terminus and the negatively charged phosphate group of DNA backbone (Figure 1(a)). The protein isolated from *Agrobacterium tumefaciens* lacks such an N-terminal and hence does not bind DNA [6]. The protein from *Mycobacterium smegmatis* can bind DNA, but duet to a positively charged C-terminus [7]. It lost the capability as soon as the C-terminus was truncated.

All of the known Dps-like proteins are composed of small subunits of about 20 kD (Table 1). They usually form an aggregate of 12 subunits with a central hollow cavity. Ferrous ions bind to the dinuclear ferroxidase sites at dimer interfaces [8–11]. Their proposed functions include oxidative detoxification, resistance to toxic electrophile and acid stress and so forth [12–14]. Many Dps proteins contain a large amount of iron. The iron sequestration of the *E. coli* Dps has also been detected [15]. A Dps-like protein, MrgA, was identified in *Bacillus subtilis* in a search for metallo-regulated genes [16]. MrgA is also inducible by starvation and protects the cell against oxidative stress [17]. Another Dps-like protein was isolated from *Listeria innocua*, and named ferritin-like protein (Flp), because it can sequester a large amount of iron in its central cavity [18]. The expression of *flp* genes in *L. innocua* and *Listeria monocytogenes* is not only inducible by both stationary phase and low iron availability [19] but also by cold shock [20]. Another Dps-like protein, the Neutrophil-activating protein (Nap), was isolated from *Helicobacter pylori* [21]. Nap was thought to mediate cell adhesion [22]. The positively charged surface makes it highly soluble. It selectively bind to acidic glycosphingolipids on human neutrophils, as well as sulphated oligosaccharides on mucin and Lewis x blood group antigen [23]. The alkaline protein surface has also been proposed to mediate DNA binding [24]. While, the Fine tangle pili (Ftp) from *Haemophilus ducreyi* has also been proposed to mediate cell adhesion [25].

We isolated a protein from *M. arborescens* SE14, and named it AAH for* N*-acyl amino acid hydrolase. *N*-acyl glutamines are plant defense elicitors in the insect saliva. AAH catalyzes the hydrolysis of the amide bond and, less efficient, the formation of the elicitor [39]. AAH also belongs to the Dps-like protein family. Since Dps-like proteins are such a structurally similar, but functionally heterogeneous group, a comparative genomic analysis was carried out to further understand its evolution, with special emphasis on the GC-rich Gram-positive Actinobacteria.

## 2. Results

**Figure 2:**
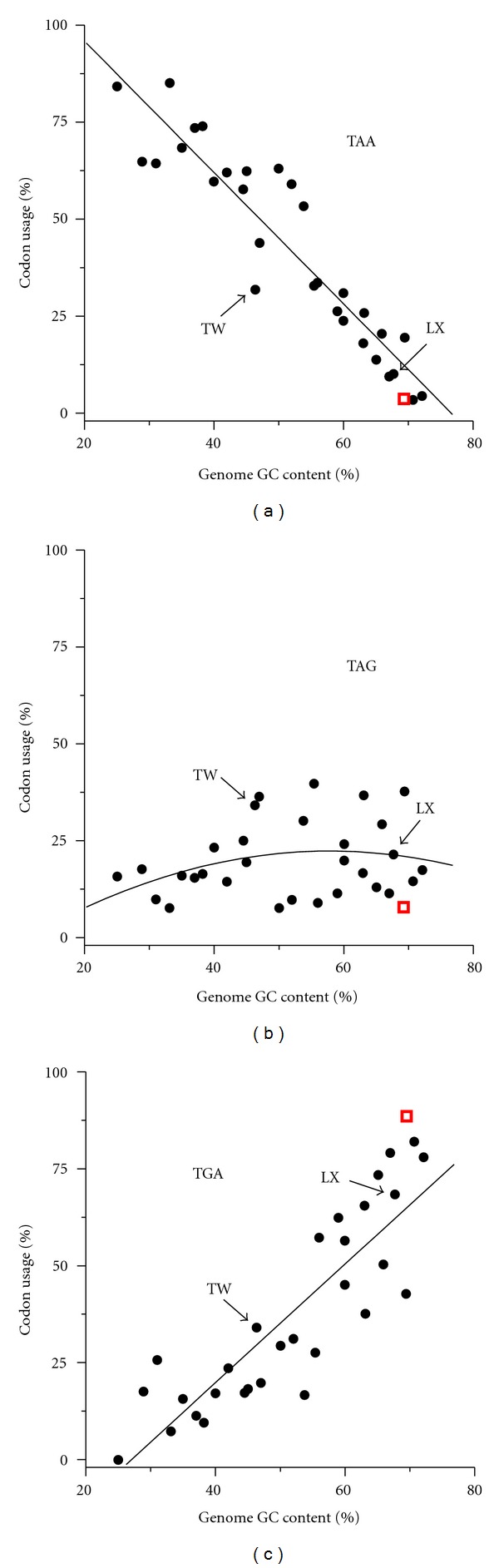
Correlation of stop codon usage and the GC content of bacterial genomes. The abundance of the stop codons were plotted against the genomic GC contents. Same kind of calculation based on the 27 predicted genes on the *M. arborescens* contig was shown as red open boxes. *T. whipplei* (TW) and *L. xyli* (LX), two species closely related to *M. arborescens*, were highlighted by arrows. The bacterial species were listed in Supplementary Table S1 available online at doi: 10.1100/2012/504905.

**Figure 3:**
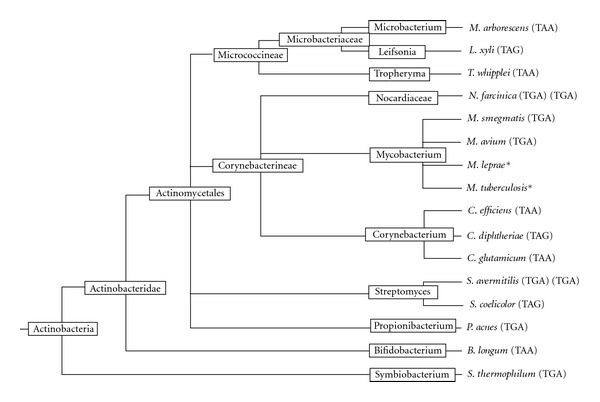
The Actinobacterial species and the stop codons used in their *dps*-like genes. The names of the classes were given in boxes at braches of the phylogenetic tree. Stop codons in *dps-*like genes were shown in brackets. Asterisks indicate no *dps*-like gene has been detected. Double brackets mean there are two copies of *dps-like *genes in the same genome.

The GC content of the *aah* gene was 67.7%, a little lower than the average value of the whole contig (69.3%) (Supplementary Table S1). The GC content of the third base of codons (GC3) of *aah* was very high (94.4%), compared to the surrounding genes. It is even higher than the GC3 of the whole *Streptomyces coelicolor* genome (92%), while the GC content of *S. coelicolor* genome is 72.1%. The GC content of the first base of codons (GC1) and of the second base of the codons (GC2) of *aah* were extremely low (Supplementary Figure S1). *aah* was the only gene on this contig using “TAA” as stop codon. When the usage of stop codons was plotted against the GC content of the genomes, a correlation emerged (Figure 2). TAA was used more often in low-GC genomes; TGA became more dominant in high-GC genomes. TAG remained at low level showed little correlation to genomic GC contents. There are exceptions, for example, the GC content of *Tropheryma whipplei* was low, but it did not use much TAA. Among the studied 14 Actinobacterial genomes, 5 *dps-*like genes used TAA, 3 used TAG, and 6 used TGA (Figure 3).

**Figure 4:**
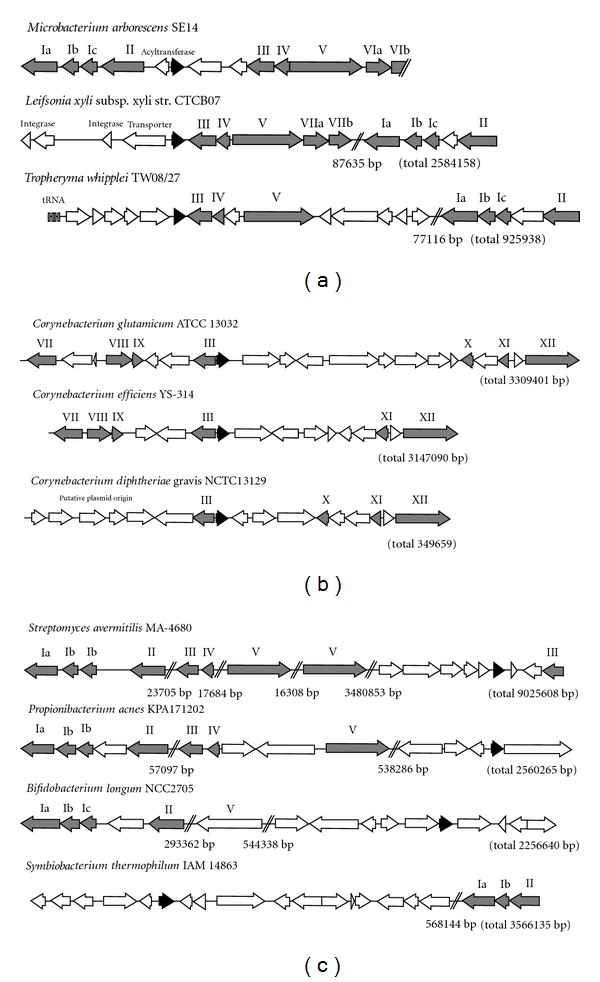
Genomic syntenies on Actinobacterial genomes surrounding the *dps-*like genes. The genomic sequences have been arbitrarily arranged so that all *dps-like *genes are transcribed to the right-hand side. Genome sizes were shown in brackets. (a) Alignment of the genomic segments of *M. arborescens* SE14, *L. xyli,* and *T. whipplei*. (b) Alignment of the genomic sequences of three Corynebacterium species. (c) Alignment of the genome of some other Actinobacteria. Regions without interesting gene were omitted and denoted as double slashes with the number of omitted bases showing underneath. The *dps-like *genes are depicted as black; other conserved genes are in gray labelled with roman numbers: Ia, ATP-dependent Clp protease ATPase; Ib, ATP-dependent Clp protease proteolytic subunits 2; Ic, ATP-dependent Clp protease proteolytic subunit 1; II, FKBP-type peptidyl-prolyl isomerase; III, formamidopyrimidine–DNA glycosylase; IV, ribose 5-phosphate isomerase; V, aminopeptidase N; VIa, ABC transporter ATPase; VIb, ABC transporter membrane component; VII, Malic enzyme; VIII, Zinc-binding dehydrogenases; IX, Methylated DNA-protein cysteine methyltransferase; X, Putative integral membrane protein; XI, MarR family transcriptional regulators; XII, Penicillin-binding protein.

A conserved genomic synteny surrounding *dps-*like genes was found in *Micrococcineae* species (Figure 4(a)). Another type of synteny was found in three *Corynebacterium *species. One gene encoding the DNA-formamidopyrimidine glycosylase presented in both syntenies (Figure 4(b)). However, the glycosylase gene and the *dps*-like gene were back to back in corynebacteria, but face to face in the *Micrococcineae* species. There were more insertion and deletion in this region on the *Corynebacterium* genomes than on the *Micrococcineae *genomes. Similar genomic context was not detected in other Actinobacteria (Figure 4(c)). AAH showed highest sequence similarity to a protein on the genome of *Leifsonia xyli *(Figure 4). Its GC content was also similar to that of this plant pathogen (Table S1).

 Coincident with the conserved genomic synteny, the three* dps*-like proteins of the *Micrococcineae *species clustered within the Actinomycetales clade (Figure 1(b)). Those proteins from the genera of the suborder *Corynebacterineae* and *Streptomycineae* formed two large clusters in the Actinomycetales group and intermingled with each other. Surprisingly, FtpA and two other proteins from *γ*-proteobacteria show high similarity to the Actinomycetales clade. This was reproducible by all three computer algorithms, namely, Neibour-joining of clustalX (NJ), NJ of Vector NTI, and maximum-likelihood of Phylip (ML). What remained to be explained is that a protein from *Bifidobacterium*, which belongs to another order, and the one from a Planctomycetes,* Rhodopirellula baltica* were also clustered to this clade.

The Dps proteins from enterobacteria grouped perfectly into one clade (Figure 1(b)). The positive N-termini were conserved in all of these proteins (data not shown). All of Dsp-like proteins from the *α*-proteobacteria had a short N-terminus (data not shown). The protein from a cyanobacterium, *Nostoc* sp. PCC 7120, was grouped into this *α*- and *γ*-proteobacteria super family. The Bacilli proteins showed higher sequence similarity than others to ferritin, which was used for rooting the tree. Proteins from spiral-shaped bacteria formed an isolated cluster. The protein from an anaerobe, *Bacteroides fragilis*, was also found in this group. Some bacteria contained two copies of *dps-like* genes, most of them showed very high sequence similarity except those two from *M. smegmatis*. On the other hand, *dps* homologs were not detected in other *Mycobacterium *species.

## 3. Discussion

Dps-like proteins widely exist in eubacteria (Table 1). The genes were proposed to have evolved from a common ancestor with bacterioferritins and eukaryotic ferritins [26]. Their reported properties can be correlated, to some extent, to their phylogeny (Figure 1(b)). The proteins from enterobacteriaceae all contain positively charged N-termini, while in *α*-proteobacteria, the termini are much shorter. Those from the spiral-shaped bacteria were surface proteins. In the Gram-positive bacilli, they mainly associated with iron sequestration. The AAH from *M. arborescens* (Actinomycetales) is, as yet, unique on the reversible hydrolase activity [39].

Genomic analysis revealed that the GC3 of the *aah* gene is significantly higher than surrounding and the *S. coelicolor* genome, though the average GC contain of the later two was much higher. The codon usage of GC-rich bacteria is always biased, resulting in a high GC3 [43]. The Sueoka theory suggests that GC1 and GC2 are under strong selection pressure, while this control is much weaker on GC3 [44]. It is, therefore, possible that the ancestor of the *aah* gene evolved from a low GC origin. It almost saturated the third position of its codons in GC content to match the host genomic environment.

Furthermore, *aah* is the only gene on the contig using TAA as stop codon. A previous study showed the high-GC thermophiles favoring TAG over TAA [45]. We analyzed 30 genomes (Figure 2) and confirmed the preference of TAA in low-GC genomes, and dominance of TGA in high-GC genomes. The slightly deviation in some species is very easy to explain, for example, *T. whipplei* that use less TAA was probably due to its evolution from a GC-rich species to a low GC environment in eukaryotic cells [46]. Other atypical species are thermophiles, halophiles, and so forth (Supplementary Table S1). They have undoubtedly encountered additional selection pressure. Among the 14 Actinobacterial *dps-*like genes studied, the stop codon TAA and TGA were equally used, which also indicates the acquisition was not an old event.

The ancestor gene might have incorporated into the *Micrococcineae *genome before the divergence of the families. The *aah* gene showed highest similarity to the proteins from *L. xyli* and *T. whipplei* (Figure 1). None of them uses TGA as stop codon (Figure 3). The genomic signature surrounding *dps-like *genes (Figure 4) seems to be a relic of the ancient incorporation event. Such conserved gene clustering has also been observed in some closely related plasmids [47]. Analysis of the *dps* gene from *B. fragilis *and *Porphyromonas gingivalis *suggests that the *dps*-like genes are present prior to the divergence of anaerobic bacterium from aerobics [31]. The ubiquity of *dps-*like genes in bacteria and the variability on functions supported the theory that the emergence of *dps-*like genes was an ancient event, and the observed sequences similarity is simply due to a common ancestry rather than common function [48].

Dps-like proteins could be grouped into four major branches. It is not a surprise to find the Gram-negative anaerobe *B. fragilis* in the Gram-positive branch, since its ancestor is the Gram-positive *Flavobacterium*/*Cytophaga *[49]. Furthermore, each major branch contains some proteobacteria. If proteobacteria is the vector dispersing the *dps* gene, the Actinomycetales would have acquired the *dps-*like gene from *γ*-proteobacteria, while *ε*-proteobacteria donated the *dps-*like gene to spirochetes. It is also possible that the intermingling of Dsp-type proteins in the dendrograph was a consequence of convergent evolution, although our data do not favour this possibility.

An earlier research revealed 50 out of 254 bacteria have more than two copies of *dps* genes [36]. We found that four species contained two copies of *dps* genes. The high similarity between the two copies suggests a recent duplication. While in *M. smegmatis*, the two copies were very different. They might have been acquired independently. On the contrary, in the other two *Mycobacterium* species, no *dps-like *gene was detected (Figure 3). Horizontal gene transfer is very limited in these bacteria [50], the *dps-like *gene might have never been acquired. After coevolution with their bacterial host over a long time, Dps proteins clearly had undergone subfunctionalization and neofunctionalization. Despite sharing a few highly conserved amino acid residues, even the ferroxidase centers vary considerably [30]. The ferroxidase centers are formed by amino acids brought together from adjacent subunits, other amino acids could also meet there to form other types of catalytic centers.

## 4. Material and Methods

Genomic DNA from *M. arborescens *SE14 was isolated as described elsewhere [39]. A cosmid library was constructed using the pWEB vector (Epicentre) according to the manufacturer's instruction. 8 positive colonies have been selected from a 1,121 colony library by PCR screening with primers afpup2: ACAGCTCGCCGATGGTCACA and afprc3: CCGTCGGCGCCGGGTATTAC. DNA insert was sheared using Standard Nebulizer (Octurno). Fragments were polished by T4 DNA and Klenow polymerases. Fragments were ligated into a pUC18 vector, and end-sequencing was achieved using BigDye Terminator Ready Reaction Kit (Applied Biosystems). Sequence data were assembled by GAP4 software (http://staden.sourceforge.net) and deposited in GenBank (accession number AY993941). Putative protein-coding sequences were predicted by the softwares Lasergene (DNAStar) and VectorNTI (Invitrogen) and then analyzed by BLAST search at NCBI (http://www.ncbi.nlm.nih.gov). Web-based software tRNAscanSE v.1.1 was used to predict tRNA [51].

The GC content was calculated with VectorNTI using a window size of 500 bp and 40 bp for the entire sequence and the *aah* gene, respectively. GC1, GC2, and GC3 were calculated by FramePlot 3.0 beta* (http://watson.nih.go.jp/) with a window size of 10 codons and step size of 1 codon. The genomic GC content and stop codon usage were calculated at website TIGR-Comprehensive Microbial Resource (http://cmr.tigr.org/tigr-scripts/CMR/CmrHomePage.cgi). To compare the genomic context, 14 Actinobacteria genomes were retrieved from NCBI and aligned manually.

The protein sequences of AAH, Dps from *E. coli*, and MrgA from *B. subtilis* were employed to BLAST at NCBI separately. Homology analysis of retrieved sequences was performed with three methods independently: (i) NJ of Vector NTI. The Guide Tree is calculated by AlignX with distance values. (ii) NJ of ClustalX 1.8 [52]. Bootstrap values were based on 1,000 replications. (iii) ML of Phylip3.67 (http://evolution.genetics.washington.edu/phylip.html). The final evolutionary tree was displayed in TreeView 1.6.6 (http://taxonomy.zoology.gla.ac.uk/rod/rod.html) and modified.

## Supplementary Material

Enclosed are the list of IDs of proteins used in Figure 1, FIGURE S1 that shows the GC profiles of the *aah* gene, and TABLE S1 that provides the information on the stop codon usage and genome GC content in some bacteria.

## Figures and Tables

**Table 1:** Reported Dps-like proteins and their proposed functions.

Name	Species	MW (kd)	DNA binding	Iron binding	Proposed function^a^	References
TpF1 (4D)	*Treponema pallidum *	19			Antigen	[3]
Dps	*Escherichia coli*	19	Yes	Yes		[1]
MrgA	*Bacillus subtilis*	16	Yes	Yes		[17]
DpsA	*Synechococcus *sp.	19.7				[26]
FtpA	*Haemophilus ducreyi*	24			Laminin binding	[25]
Flp	*Listeria innocua*	18		Yes		[18]
Flp	*Listeria monocytogenes*	18			Cold shock protein	[20]
NapA	*Helicobacter pylori*	17	No	Yes	Cell adhesion	[22, 23, 27]
Dpr	*Streptococcus mutans*	19.7		Yes		[28, 29]
Dpr	*Streptococcal suis*	19.6	No	Yes	Galactose adhesion	[30]
Dps	*Bacteroides fragilis*	17.9				[31]
Dlp-1	*Bacillus anthracis*	16.9	No	Yes		[10]
Dlp-2	*Bacillus anthracis*	16.7	No	Yes		[10]
MrgA	*Staphylococcus aureus*	16.7				[32]
Dps	*Campylobacter jejuni*	17.3	No	Yes		[33]
Dps1	*Mycobacterium smegmatis*	21.6	Yes	Yes		[34, 35]
Dps2	*Mycobacterium smegmatis*	17.8	Yes	Yes		[36]
Dps	*Agrobacterium tumefaciens*	18.6	No	Yes		[6]
Dps	*Bacillus brevis*	16.3	Yes	Yes		[11]
Dps	*Porphyromonas gingivalis*	19				[37]
Dps	*Proteus vulgaris*					[38]
Dps	*Serratia marcescens*					[38]
Dps	*Salmonella enterica*					[38]
Dps	*Klebsiella pneumoniae*					[38]
Dps	*Enterobacter cloacae*					[38]
Dps	*Citrobacter freundii*					[38]
AAH	*Microbacterium arborescens*	17.1	No	Yes	Reversible hydrolysis	[39]
Dps1	*Deinococcus radiodurans*	23.0	Yes	Yes		[40]
Dps2	*Deinococcus radiodurans*	26.1	Yes	Yes		[41]
Dps	*Nostoc *sp.	20.1	No	Yes		[42]

Blank means no report. ^a^Proposed function other than oxidative detoxification, iron oxidation, and storage.
