# Supplementary material for: Coevolution of aah: A dps-Like Gene with the Host Bacterium Revealed by Comparative Genomic Analysis
Source: ScientificWorldJournal. 2012 Feb 1;2012:504905. doi: 10.1100/2012/504905 (PMC3289904; doi:10.1100/2012/504905)
Supplement: Supplementary file 1 — Enclosed are the list of IDs of proteins used in Figure 1, FIGURE S1 that shows the GC profiles of the aah gene, and TABLE S1 that provides the information on the stop codon usage and genome GC content in some bacteria. [file 504905.f1.pdf]

The IDs of proteins used in Figure 1:

ACIAD1205-*A.* sp. ADP1 (CAG68082); AAH-*M. arborescens* (AAV65949); all1173-*N.* sp. PCC7120 (BAB73130); BL0618-*B. longum* (NP_695805); BR2149-*B. suis* (AAN31039); CE2833-*C. efficiens* (BAC19643); Cgl2999-*C. glutamicum* (BAC00393); CJE1705-C. jejuni (YP_179683); DIP2303-*C. diphtheriae* (CAE50826); Dpl-1-*B. anthracis* (AAP28955); Dpl-2-*B. anthracis* (AAP25903); Dpr-*S. mutans* (BAA96472); DpsA-*S. elongatus* (YP_401188); Dps-*A. tumefaciens* (NP_533149); Dps-*B. brevis* (1N1QA); Dps-*B. fragilis* (BAD48082); Dps-*C. feundii* (AAO47739); Dps-*E. cloacae* (AAO41729); Dps-*E. coli* (NP_415333); Dps-*K. cryocrescens* (AAO41728); Dps-*K. pneumoniae* (AAO41730); Dps-*P. vulgaris* (AAO47740); Dps-*S. aureus* (NP_372663); Dps-*S. marcescens* (AAO47741); Dps-*S. flexneri* (AAN42397); Dps- *M. smegmatis* (AAL40885); DpsA-*S. elongatus* (AAA86318); ECA2767-E. carotovora (CAG75667); Flp-*L. innocua* (P80725); FtpA-*H. ducreyi* (AAP95079); IL0075-*I. loihiensis* (AAV80918); GOX0707-*G. oxydans* (AAW60484); Lxx08790-*L. xyli* (YP_061879); MAP3560-*M. avium* (AAS06110); MrgA-*B. subtilis* (NP_391178); MS0670-*M. succiniciproducens* (AAU37277); NapA-*H. pylori* (AAD07308); nfa13000-*N. farcinica* (YP_117509); nfa43820- *N. farcinica* (YP_120595); PPA2134-*P. acnes* (AAT83841); RB4433-*R. baltica* (CAD73784); SAV1605-*S. avermitilis* (BAC69316); SAV2504-*S. avermitilis* (BAC70215); SCO0596-*S. coelicolor* (CAB61290); STH866-S. thermophilum (YP_074695); STM0831-*S. typhimurium* (NP_459808); TpF1-*T. pallidum* (P16665); TW375-*T. whipplei* (NP_789308); y1677-*Y. pestis* (NP_668995); Ferritin-*E. coli* (AAC74975)

FIGURE S1. The GC profiles of the *aah* gene.

A, the GC distribution on the whole cotig. The location of the *aah* gene is indicated by a black arrow. B, the GC content of the whole *aah* coding region. The gene is diagrammed as a hollow arrow. C, Plots of GC content at different base positions of the codons. Solid curve is GC3; broken curve is GC1; dotted curve is GC2. The broken straight line is the average GC content of the ORF with value shown on the y axis.

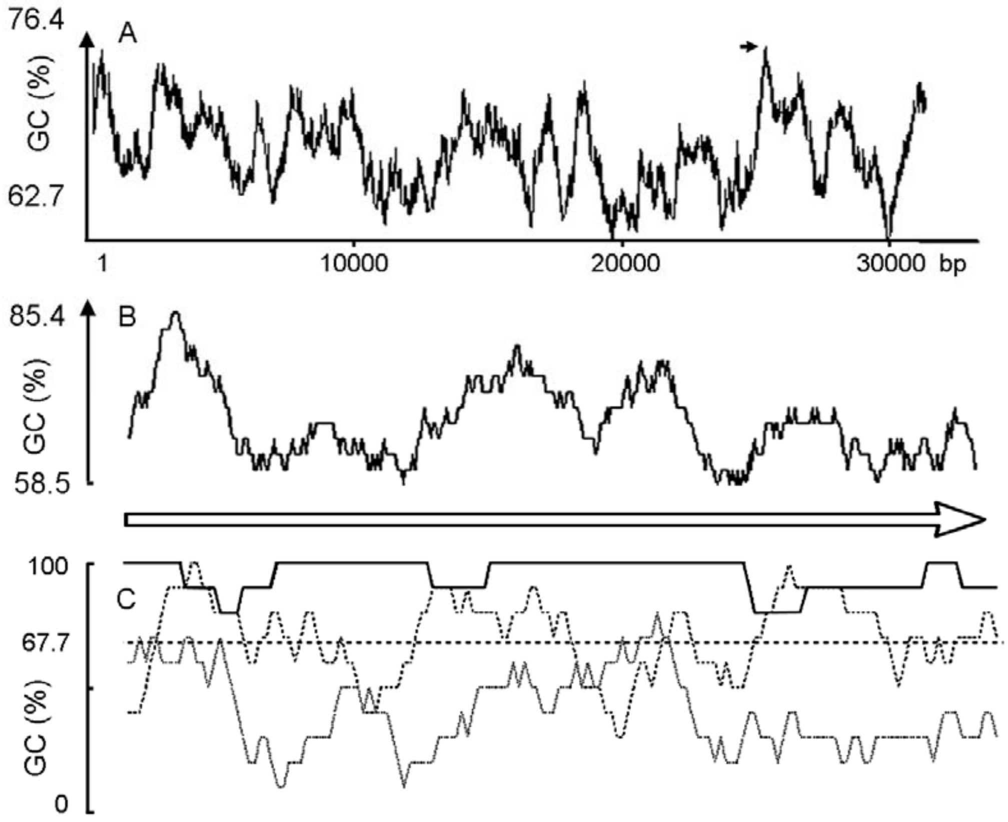

TABLE S1. The stop codon usage and genome GC content in some bacteria.

Species [a]	GC content (%)	ORF counted	Stop codon usage (%) [b]		
			TAA	TAG	TGA
Mycoplasma mobile 163K	25.0	635	84.25	15.75	0
Rickettsia typhi wilmington	28.9	835	64.79	17.60	17.60
Campylobacter jejuni RM1221	31.0	1840	64.46	9.84	25.71
Methanococcus maripaludis S2	33.1	1714	85.06	7.58	7.35
Bacillus anthracis Ames	35.0	5365	68.33	16.01	15.66
Staphylococcus aureus MW2	37.0	2632	73.44	15.35	11.21
Haemophilus ducreyi 35000HP	38.2	1717	74.02	16.48	9.49
Streptococcus pneumoniae R6	40.0	2043	59.72	23.20	17.08
Bacillus subtilis 168	42.0	4098	62.05	14.42	23.52
Lactobacillus plantarum WCFS1	44.5	3009	57.76	25.06	17.18
Vibrio vulnificus YJ016	45.0	3261	62.43	19.38	18.18
***Tropheryma whipplei* TW08/27**	46.3	780	31.79	34.23	33.97
Synechocystis sp. PCC6803	47.0	3163	43.82	36.36	19.82
Escherichia coli K12-MG1655	50.0	4285	63.08	7.61	29.31
Salmonella enterica serovar Typhi CT18	52.0	4387	59.04	9.78	31.18
Corynebacterium glutamicum ATCC 13032	53.8	3057	53.32	30.06	16.62
Synechococcus elongatus PCC 7942	55.4	2611	32.82	39.64	27.54
Chlorobium tepidum TLS	56.0	2254	33.72	9.01	57.28
Agrobacterium tumefaciens str. C58	59.0	2785	26.25	11.45	62.30
Desulfovibrio desulfuricans G20	60.0	3775	30.86	24.08	45.06
Bifidobacterium longum NCC2705	60.0	1729	23.71	19.84	56.45
Thiobacillus denitrificans ATCC 25259	63.0	2827	17.97	16.63	65.41
Corynebacterium efficiens YS-314	63.1	2950	25.8	36.68	37.53
Azoarcus sp. EbN1	65.1	4115	13.68	12.95	73.37
Halobacterium sp. NRC-1	65.9	2058	20.46	29.2	50.34
Pseudomonas aeruginosa PAO1	67.0	5565	9.45	11.48	79.07
***Leifsonia xyli* CTCB07**	67.7	2030	10.1	21.48	68.42
***M. arborescens* SE14 - 10D5**	69.3	27	3.70	7.41	88.89
Thermus thermophilus HB27	69.4	1982	19.48	37.74	42.79
Nocardia farcinica IFM 10152	70.7	5722	3.43	14.59	81.98
Streptomyces coelicolor A3(2)	72.1	7493	4.46	17.52	78.02

[a]. All data are the result of analying the whole genome, except *M. arborescens* SE14. All non-chromosome sequences such as plasmids had been omitted even if they had been deposited. Bold words are for highlighting purposes.

[b]. In Mycoplasma species, “TGA” is used to encode tryptophan instead of stop signal, *e.g* there are 1756 “TGA” coding Tryptophan in the genome in *Mycoplasma mobile* 163K.
